# *Acanthamoeba* Infection and Nasal Rinsing, United States, 1994–2022

**DOI:** 10.3201/eid3004.231076

**Published:** 2024-04

**Authors:** Julia C. Haston, Chelsea Serra, Erin Imada, Emalee Martin, Ibne Karim M. Ali, Jennifer R. Cope

**Affiliations:** Centers for Disease Control and Prevention, Atlanta, Georgia, USA (J.C. Haston, C. Serra, E. Imada, E. Martin, I.K.M. Ali, J.R. Cope);; Public Health Institute and CDC Global Health Fellowship Program, Oakland, California, USA (C. Serra)

**Keywords:** *Acanthamoeba*, nasal rinsing, amebae, tap water, granulomatous amebic encephalitis, parasites, United States

## Abstract

We describe 10 patients with nonkeratitis *Acanthamoeba* infection who reported performing nasal rinsing before becoming ill. All were immunocompromised, 7 had chronic sinusitis, and many used tap water for nasal rinsing. Immunocompromised persons should be educated about safe nasal rinsing to prevent free-living ameba infections.

*Acanthamoeba* spp. are free-living amebae (FLA) found worldwide in soil and many types of water, including lakes, rivers, and tap water (*1*–*3*). *Acanthamoeba* amebae can cause keratitis, which is an infection of the eye that does not spread to other parts of the body. However, they can also cause a variety of severe human infections, including granulomatous amebic encephalitis (GAE), an infection of the central nervous system, as well as cutaneous disease, rhinosinusitis, pulmonary disease, osteomyelitis, and disseminated infections (*1*). *Acanthamoeba* amebae cause disease when they enter the body through the eyes, broken skin, or respiratory tract (*1*). The ameba is known to be an opportunistic pathogen, and persons at highest risk of infection include those with a history of solid organ or stem cell transplant, cancer (specifically hematologic cancers), HIV, or diabetes mellitus (*4*,*5*). Nonkeratitis *Acanthamoeba* infections are rare, affecting only 3–12 persons annually in the United States; however, 82% of cases are fatal (*5*).

Because *Acanthamoeba* amebae are ubiquitous in the environment, the source of infection is often unknown, and identifying prevention strategies is challenging. However, performing safe nasal rinsing may be one way to prevent *Acanthamoeba* infection. Nasal rinsing is the practice of irrigating the sinuses for either health or religious purposes (e.g., ritual ablution) (*6*). There are a variety of ways nasal rinsing can be performed, including through the use of a device (e.g., a neti pot or squeeze bottle) or by using cupped hands to hold water. Nasal rinsing can provide health benefits, but it can also introduce pathogens, particularly if unsterile water is used (*7*). Nasal rinsing with tap water has been associated with infections caused by FLA, including *Naegleria fowleri* and *Acanthamoeba* (*8*,*9*). A recent study showed that nearly two thirds of US adults think tap water is safe for nasal rinsing (*7*). Our case study describes clinical features and nasal rinsing behaviors of US patients with *Acanthamoeba* infections who performed nasal rinsing. Our results underscore the importance of increasing awareness about safe nasal rinsing.

## The Study

We used the Centers for Disease Control and Prevention (CDC) FLA database to identify US patients with laboratory-confirmed nonkeratitis *Acanthamoeba* infections who reported nasal rinsing before the onset of symptoms. We analyzed demographic and clinical characteristics for each case. We followed the process of Haston et al. in creating the algorithm used to classify disease manifestations (*5*). Our study protocol was reviewed by CDC and our research was conducted consistent with applicable federal law and CDC policy (see, e.g., 45 C.F.R. part 46.102(I) (*2*), 21 C.F.R. part 56; 42 U.S.C. §241(d); 5 U.S.C. §552a; 44 U.S.C. §3501 et seq.).

Ten patients reported nasal rinsing before their *Acanthamoeba* diagnoses. Infections occurred during 1994–2022, but 9 cases occurred in the past decade. The median age of patients was 60 years (range 32–80 years) (Table 1). Seven patients were male, and 3 were female. All 10 patients had >1 immunocompromising condition, most commonly cancer (Table 2). Four of 5 patients with cancer had chronic lymphocytic leukemia. Two patients had HIV with CD4 counts <100 cells/mm^3^ at the time of *Acanthamoeba* diagnosis, meeting criteria for AIDS. Seven patients survived, which is unexpectedly high considering the typical fatality rate for *Acanthamoeba* infection. Most patients were diagnosed by PCR, including 6 of 7 survivors.

**Table 1:** Demographic and clinical characteristics of 10 patients with *Acanthamoeba* infection who performed nasal rinsing, United States, 1994–2022

Demographic	Value
Median age (range), y	60 (32–80)
Sex	
M	7
F	3
Race	
White	5
Black	2
Asian/Pacific Islander	1
Unknown	2
Ethnicity	
Hispanic	1
Non-Hispanic	4
Unknown	5
Disease manifestation, confirmed or suspected†	
Rhinosinusitis	9
GAE	6
Cutaneous	6
Osteomyelitis	3
Pulmonary	1
Endophthalmitis	1
Diagnostic method†	
PCR	7
Histopathology	6
Immunohistochemical staining	5
Indirect immunofluorescence	2
Specimens tested†	
Skin	6
Brain	4
Sinus	4
Bone	3
Genotype	
T4	4
T1	1
Unknown	5
Underlying conditions†	
Chronic sinusitis	7
Cancer‡	5
Chronic kidney disease	5
HIV/AIDS	2
Solid organ transplant	2
Stem cell transplant	1
Microscopic Polyangiitis	1
Outcome	
Survived	7
Died	3

*Values are no. (%) patients except as indicated. GAE, granulomatous amebic encephalitis. †Some patients may have had multiple disease manifestations, multiple diagnostic methods, or multiple comorbid conditions. ‡Four chronic lymphocytic leukemia, 1 acute myeloid leukemia.

**Table 2:** Nasal rinsing practices, medical histories, and disease presentations of 10 patients with *Acanthamoeba* infections who performed nasal rinsing, United States1994–2022*

Year and reference	Age, y/ sex	State	Reason for and type of nasal rinsing	Relevant medical history	Initial symptom(s)	Signs and symptoms	Disease manifestations	Outcome
1994, Murakawa et al. (*10*)	45/M	CA	Chronic sinusitis; electric nasal irrigator with tap water	HIV/AIDS (CD4: 26 cells/mm^3^); co-infections: PCP, *Candida* esophagitis, perianal herpes simplex	Skin lesions	Pruritic, painful, erythematous papulonodular, ulcerative lesions on the extremities, face, and genitalia; seizures; pansinusitis on MRI; osteolytic lesions	Cutaneous, rhinosinusitis, osteomyelitis, suspected GAE	Died
2015, Brondfield et al. (*11*)	60/F	CA	Chronic sinusitis; nasal irrigation using tap water for several months before symptom onset	Heart transplant (nonischemic cardiomyopathy); chronic kidney disease	Rhinorrhea, sinus pressure, epistaxis	Acute sinusitis and development of septal mass; subcutaneous nodules of left flank and extremities; pathologic fracture of right metatarsal bone underlying a nodule	Cutaneous, rhinosinusitis, osteomyelitis	Survived
2015, Breland et al. (*12*), Winsett et al. (*13*)	36/F	TX	Chronic sinusitis; neti pot with tap water for 2 wks before symptom onset	Kidney transplant (ESRD due to diabetes mellitus)	Skin lesions	Painful nodules on bilateral upper extremities, face, and back, spreading to lower extremities and trunk; later, fever, dyspnea, nausea, vomiting, and knee pain with cortical destruction in the proximal fibula; abnormal lung imaging	Cutaneous, osteomyelitis, suspected rhinosinusitis, suspected pulmonary	Survived
2016, CDC, unpub. data†	59/F	MD	Ritual ablution; no further information available	HIV/AIDS (CD4: 97 cells/mm^3^); co-infections: *Salmonella* bacteremia	Headache and blurred vision	Headache, blurred vision, multiple brain lesions; later, fever, disorientation/altered mental status, ataxia, weakness, cranial nerve deficits	GAE	Died
2017, Voshtina et al. (*14*)	73/M	WI	Unknown reason; neti pot using sterile water 1×/wk (submerged filled neti pot in warm tap water to heat)	Cancer: CLL	Focal seizure	Focal seizure and headache; later, fever, lethargy, altered mental status, cranial nerve deficits, cerebral herniation	GAE, suspected rhinosinusitis	Died
2017, CDC, unpub. data†	64/M	OH	Chronic sinusitis; nasal irrigation with undistilled tap water	Cancer: CLL	Sinusitis, skin lesions	Sinusitis and tender, erythematous skin lesions on lower extremities; later, altered mental status, headaches, photophobia, phonophobia, hallucinations, cranial nerve deficits, declining mental status	GAE, cutaneous, suspected rhinosinusitis	Survived
2018, CDC, unpub. data†	32/M	TX	Chronic sinusitis and epistaxis for 2 y; squeeze bottle with saline nasal spray	Microscopic polyangiitis; pauci-immune glomerulonephritis; renal failure	Seizure	Seizure; weakness, paresthesias, fatigue; later, meningismus, altered mental status, fever, aphasia, dysphagia, facial numbness, hemiparesis, cranial nerve deficits, acute respiratory failure	GAE, suspected rhinosinusitis	Survived
2020, CDC, unpub. data†	46/M	TX	Ritual ablution 5×/d	Cancer: AML; stem cell transplant ×2	Neutropenic fever	Fever; later, facial pain, papular and nodular erythematous skin lesions on lower extremities, sinusitis, endophthalmitis, brain lesions on MRI	Cutaneous, suspected GAE, suspected rhinosinusitis, suspected endophthalmitis	Survived
2021, CDC, unpub. data†	66/M	MD	Chronic sinusitis after multiple surgeries for orbital fracture and deviated septum; squeeze bottle 4×/d for years	Cancer: CLL; chronic kidney disease; multiple reconstructive surgeries following orbital fracture	Sinusitis; skin lesion	Sinusitis; progressive, necrotizing facial lesion	Cutaneous, rhinosinusitis	Survived
2022, CDC, unpub. data†	80/M	FL	Chronic sinusitis and nasal surgery; squeeze bottle daily for 3 y (water source unknown)	Cancer: CLL; chronic kidney disease	Headaches related to sinus disease	Chronic headaches; nasal congestion, postnasal drip; occasional nausea and vomiting	Rhinosinusitis	Survived

*AML, acute myeloid leukemia; CDC, Centers for Disease Control and Prevention. CLL, chronic lymphocytic leukemia; ESRD, end-stage renal disease; GAE, granulomatous amebic encephalitis; MRI, magnetic resonance imaging; PCP, *Pneumocystis jirovecii* pneumonia; †Cases from the CDC free-living amebae database.

Including both confirmed and suspected *Acanthamoeba* disease manifestations, 9 patients were diagnosed with rhinosinusitis, 6 had GAE, 6 had cutaneous disease, and 3 had osteomyelitis. Eight patients had evidence of disseminated disease, and all of those 8 patients were diagnosed with rhinosinusitis. At least 7 patients had a history of chronic sinusitis, so there may have been a delay in identifying acute sinus symptoms, enabling dissemination to other organ systems in these patients.

The high percentage of patients presenting with skin or sinus manifestations may have contributed to the unusually high survival rate in this small cohort, because treatment may have been initiated at earlier stages of disease for some. Previous studies have shown that GAE is associated with poor prognosis; survival rates are <7%. In this cohort, 3 patients with confirmed or suspected GAE died and 3 survived. Of the GAE survivors, 2 developed skin lesions before central nervous system symptoms.

Seven patients reported nasal rinsing for relief of chronic sinusitis symptoms, 2 performed ritual ablution, and 1 did not report a reason for nasal rinsing. At least 4 patients reported using tap water for nasal rinsing, and 1 other patient reported using sterile water but then submerging the device in tap water. A water source was not reported for the other 5 patients. Of the 6 patients who described nasal rinsing devices, 3 used squeeze bottles, 2 used neti pots, and 1 used an electric nasal irrigator. Duration and frequency of nasal rinsing varied. One patient developed symptoms after only 2 weeks of nasal rinsing, whereas others had been nasal rinsing for years. The frequency of nasal rinsing ranged from 1 time per week to 5 times per day.

## Conclusions

In these 10 case-patients with invasive *Acanthamoeba* infection, nasal rinsing may have been the transmission route. Duration and frequency of nasal rinsing behaviors varied, but most patients had been rinsing for months or even years. Whereas amebae could theoretically be introduced during any rinsing encounter, the risk of infection likely increases over time with continued exposure. At least half of the patients in this case series used tap water in their nasal rinsing practices. Even though *Acanthamoeba* and other biofilm-associated amebae have been detected in >50% of US tap water samples, a recent study reported that 33% of US adults believe that tap water is sterile, and 62% believe it to be safe for rinsing sinuses (*3*,*7*). Educating against the use of unboiled tap water for nasal rinsing may be effective in preventing invasive *Acanthamoeba* infections, particularly among immunocompromised hosts.

Most patients in this case series performed rinsing for chronic sinusitis and likely had damaged mucosal tissue because of their underlying illnesses, which may have increased the risk for infection. Furthermore, all patients were immunocompromised. Some patients demonstrated signs of acute or worsening sinusitis on initial examination, but others demonstrated only skin lesions or neurologic symptoms. Of note, the 2 patients who performed ritual ablution did not initially have sinus symptoms, which suggests that patients with underlying sinus disease may be more likely to initially have symptoms of *Acanthamoeba* rhinosinusitis develop compared with those who do not.

The first limitation of our study is that causation cannot be determined using these data. Nasal rinsing was not definitively determined to be the route of transmission for any case. Second, survival beyond the date of report cannot be confirmed. The nature of passive case surveillance data does not allow for patient follow-up. Finally, information regarding nasal rinsing practices were limited for some cases. The small number of cases did not enable thorough assessment of practices that may be considered to increase risk for infection.

All healthcare providers caring for immunocompromised persons should educate their patients about *Acanthamoeba* infections, including how to recognize symptoms and how to practice safe nasal rinsing. CDC recommendations for performing safe nasal rinsing include using boiled, sterile, or distilled water (*6*). If tap water is used, it should be boiled for a minimum of 1 minute, or 3 minutes in elevations >1,980 meters, and cooled before use (*6*). For diagnostic support and treatment recommendations, CDC offers a 24/7 Free-Living Ameba Consultation Service. Healthcare providers can call the CDC Emergency Operations Center at (770) 488–7100 for a consultation for any confirmed or suspected *Acanthamoeba* infection.
